# Objective coding of content and techniques in workplace-based supervision of an EBT in public mental health

**DOI:** 10.1186/s13012-017-0708-3

**Published:** 2018-01-24

**Authors:** Shannon Dorsey, Suzanne E. U. Kerns, Leah Lucid, Michael D. Pullmann, Julie P. Harrison, Lucy Berliner, Kelly Thompson, Esther Deblinger

**Affiliations:** 1 Department of Psychology, University of Washington, Guthrie Hall, Box 351525, Seattle, WA 98195 USA; 2 University of Denver, Graduate School of Social Work, Craig Hall, Room 471, 2148 S. High St, Denver, CO 80208 USA; 3 Division of Public Behavioral Health and Justice Policy, University of Washington School of Medicine, 2815 Eastlake Ave E, Suite 200, Seattle, WA 98102 USA; 4 Harborview Center for Sexual Assault and Traumatic Stress, University of Washington School of Medicine, 401 Broadway, Suite 2027, Seattle, WA 98122 USA; 5 CARES Institute, Rowan University School of Osteopathic Medicine, 42 E. Laurel Road, UDP, Suite 1100, Stratford, NJ 08084 USA

**Keywords:** Clinical supervision, Behavioral health, Public mental health, Children’s mental health, Evidence-based practice, Evidence-based treatment, Objective coding

## Abstract

**Background:**

Workplace-based clinical supervision as an implementation strategy to support evidence-based treatment (EBT) in public mental health has received limited research attention. A commonly provided infrastructure support, it may offer a relatively cost-neutral implementation strategy for organizations. However, research has not objectively examined workplace-based supervision of EBT and specifically how it might differ from EBT supervision provided in efficacy and effectiveness trials.

**Methods:**

Data come from a descriptive study of supervision in the context of a state-funded EBT implementation effort. Verbal interactions from audio recordings of 438 supervision sessions between 28 supervisors and 70 clinicians from 17 public mental health organizations (in 23 offices) were objectively coded for presence and intensity coverage of 29 supervision strategies (16 content and 13 technique items), duration, and temporal focus. Random effects mixed models estimated proportion of variance in content and techniques attributable to the supervisor and clinician levels.

**Results:**

Interrater reliability among coders was excellent. EBT cases averaged 12.4 min of supervision per session. Intensity of coverage for EBT content varied, with some discussed frequently at medium or high intensity (exposure) and others infrequently discussed or discussed only at low intensity (behavior management; assigning/reviewing client homework). Other than fidelity assessment, supervision techniques common in treatment trials (e.g., reviewing actual practice, behavioral rehearsal) were used rarely or primarily at low intensity. In general, EBT content clustered more at the clinician level; different techniques clustered at either the clinician or supervisor level.

**Conclusions:**

Workplace-based clinical supervision may be a feasible implementation strategy for supporting EBT implementation, yet it differs from supervision in treatment trials. Time allotted per case is limited, compressing time for EBT coverage. Techniques that involve observation of clinician skills are rarely used. Workplace-based supervision content appears to be tailored to individual clinicians and driven to some degree by the individual supervisor. Our findings point to areas for intervention to enhance the potential of workplace-based supervision for implementation effectiveness.

**Trial registration:**

NCT01800266, Clinical Trials, Retrospectively Registered (for this descriptive study; registration prior to any intervention [part of phase II RCT, this manuscript is only phase I descriptive results])

**Electronic supplementary material:**

The online version of this article (10.1186/s13012-017-0708-3) contains supplementary material, which is available to authorized users.

## Background

Clinical supervision is an implementation strategy defined as “providing clinicians with ongoing supervision focusing on the innovation” [1]. Reviews of mental health provider training in evidence-based treatments (EBT) indicate that clinical supervision following training is required to positively impact provider behavior [2, 3]; “*there does not seem to be a substitute* for expert consultation, supervision, and feedback for improving skills and increasing adoption” [3]. Studies suggest that clinical supervision may be even more important than the type of training for adherence and competency [4–6]. Yet, implementation science research has infrequently focused on clinical supervision and supervisor-level practices [7].

Clinical supervision in Powell and colleagues’ compilation of implementation strategies [1] can encompass two related but distinct activities: expert clinical consultation, provided by established experts external to the organization [8], and workplace-based supervision, provided by supervisors employed by the organization. In the growing literature focused on expert consultation, EBT-focused consultation following in-person training positively impacts provider behavior [9, 10] and clinician competency [10], with higher doses of consultation predicting higher competency (e.g., [4]). Recent studies have coded expert consultation for content and techniques [11] and examined different methods of providing consultation (e.g., group vs. individual; phone vs. live video coaching) and their association with provider [12] and client outcomes [13]. Other studies of expert consultation have examined whether use of active learning techniques, including supervisor modeling and clinician behavioral rehearsal, predict provider-level outcomes including clinician fidelity, skill, and knowledge [11, 14, 15].

Research on expert consultation has outpaced research focused on workplace-based supervision of EBT. In children’s community mental health, weekly workplace-based clinical supervision was reported by organizations as a highly common infrastructure support [16]. As such, it may offer a naturally occurring, relatively low-cost implementation strategy to support EBT in community settings [17, 18], where financial challenges [19] may preclude ongoing use of expert consultation, potentially threatening EBT sustainment [20, 21]. Some EBTs and/or implementation efforts have required that workplace-based supervisors be trained in the EBT and have provided some supervisor-specific training [22–25], presumably to harness supervision as an implementation support. However, to our knowledge, research has not objectively examined what happens in workplace-based supervision of EBT following clinician and supervisor training in EBT.

Accurso and colleagues examined the content of routine workplace-based supervision, looking for potential concordance with EBT content [26]. Using self-report, they found that the most common supervision functions were case conceptualization and interventions. Coverage of EBT-consistent content elements (assigning/reviewing homework, positive reinforcement) was brief, and use of supervision techniques common in efficacy trials [27] including video/audio tape review and fidelity monitoring was infrequent (13% and 4.6%, respectively). Building on their study, our team examined some of these same questions, also using self-report, in the context of a statewide EBT implementation effort, in which all participating supervisors had been trained in an EBT [18]. Our results indicated that nearly 70% of supervision was clinically focused (vs. non-clinical functions, including administrative), but only about half of the clinical time was spent on case conceptualization and interventions (about 20 min of a typical supervision hour).

A few studies have intervened on workplace-based supervision. The most rigorous work has focused on Multisystemic therapy (MST) [28] and audit and feedback [29]. In a large study (45 organizations; nearly 500 clinicians), supervisors were trained in a manualized supervision model for MST [30]. Adherence to aspects of the supervision model (i.e., including a focus on MST treatment principles) predicted clinician adherence to MST and client outcomes [31]. A small quasi-experimental study with psychiatric nurses found that supervisors who were trained to include gold standard elements had a positive impact on provider knowledge, attitudes, and client outcomes [32]. Looking to healthcare more broadly, the specific technique of audit and feedback has a robust body of evidence for positively impacting provider behavior and skill [29].

More supervision-level intervention studies focused on EBT are needed, given existing demands on workplace-based supervision to meet a wide range of needs beyond clinical and EBT support [18, 33]. However, to inform these efforts, a better understanding of what happens in workplace-based supervision following EBT training is necessary. Based on what we know from efficacy trials, expert consultation, and the limited workplace-based EBT supervision research, if supervision is to be used to support EBT, it should include a “sufficient” dose of EBT-focused coverage [27] and active learning techniques from efficacy trials, here forward referred to as “gold standard” techniques. However, workplace-based supervisors cover a wide variety of other clinical (e.g., crisis and case management) and non-clinical areas (e.g., administrative) with clinicians who have high caseloads [18, 34]. These differences may present challenges (e.g., limited available time) for integration of EBT coverage into supervision and for use of gold standard supervision techniques.

The goal of our study was to objectively describe supervision strategies within a state-funded EBT initiative [17, 23] representative of other statewide initiatives for the same child- and adolescent-focused EBT [24]. We were interested in characterizing supervision provided by workplace-based supervisors, including time per case, content and techniques used (many of which overlap with other child and adult EBT), and temporal focus of supervision. Additionally, given literature suggesting that clinician- and client-level characteristics are associated with what happens in therapy sessions [35, 36], we were interested in exploring a parallel for supervision sessions, specifying if strategy use is driven more by supervisors, clinicians, or both. Therefore, we examined the proportion of variance in content and technique intensity accounted for at the supervisor- and clinician-level, as understanding association generally by level can inform future investigations of specific characteristics at each level.

## Methods

Data come from a two-phase NIMH-funded study of workplace-based clinical supervision of an EBT with primary aims of (1) describing “baseline” supervision strategies (phase I), (2) evaluating the effects of two different supervision packages that incorporate gold standard elements from efficacy and effectiveness trials on clinician fidelity and client outcomes (via randomized controlled trial [RCT]; phase II), and (3) testing fidelity as a mediator of supervision condition and client outcomes [17].

The current study addresses aim 1 using data from phase I.

The study builds on a statewide EBT training initiative. In 2007, Washington State began modestly funding training in Trauma-focused Cognitive Behavioral Therapy (TF-CBT) for public mental health organizations [37]. Since 2009, training also included CBT for depression, anxiety, and behavior problems, with 100–250 trainees per year. Trainings were 2 (prior to 2009) or 3 days in duration (after 2009, due to expanded content). Organizations could send trainees every year to address growth-related needs and attrition. Trainees were expected to participate in 6 months of post-training expert consultation via 1-h conference calls, held twice a month. Organizations were required to have at least one supervisor also complete initiative expectations. Supervisor-specific post-training supports were available via optional monthly technical assistance calls and a yearly one-day supervisor training. As of 2015, 83% of the 109 public mental health organizations had participated in at least one training.

### Procedure

Procedures were approved by the Washington State Institutional Review Board. The study team identified organizations that participated in the EBT initiative, were implementing TF-CBT, and had at least one TF-CBT-trained supervisor. We provided supervisors and senior leaders with detailed study descriptions. Eligible clinicians were identified by training registration lists (trained in TF-CBT; supervised by one of the participating supervisors) and invited by our study team to participate. Supervisors who chose to participate informed the study team about which eligible clinicians in their organization were under their direct supervision. Informed consent was obtained prior to participation. In phase I, 72% of the organizations (18 of 25, 76.7% of the supervisors (33 of 43), and 76% of the clinicians (95 of 125) approached consented to participate. In phase I, supervisors and clinicians completed online baseline surveys in September 2012 prior to a required 2-day TF-CBT booster and study procedures training. Clinicians and supervisors received $30 each for completing the baseline survey; participating organizations received $3000 at the end of the study.

During phase I (October 2012–September 2013), participating supervisors were asked to audio-record weekly individual supervision of TF-CBT cases with participating clinicians and send recordings to the study team. Informal supervision, occurring outside of designated supervision time, was not recorded. Recordings were saved on study-provided, password-protected tablets and transferred to the study team using a cloud-based server compliant with the Health Insurance Portability and Accountability Act of 1996.

### Participants

#### Supervisor participants

Table 1 provides demographic information for all participants. Participants were located in 18 public mental health organizations in 23 offices throughout Washington State. Criteria for study inclusion were receiving TF-CBT-specific training as part of the EBT initiative and being a current supervisor of two or more clinicians who were eligible to participate. There were no exclusionary criteria. Thirty-three supervisors were enrolled in phase I; this study analyzed data from 28 supervisors (85%; from 17 of the 18 organizations) who submitted recordings of individual supervision sessions (three supervisors did not submit recordings [two of whom left their organizations within 2 months]; two others submitted group recordings that could not be coded).

**Table 1:** Demographics of supervisors and clinicians who submitted audiotaped supervision sessions

Variable	Supervisor (*n* = 28)		Clinician (*n* = 70)	
	*n*	%	*n*	%
Female	18	64.3	61	87.1
Race/ethnicity				
White/Caucasian	26	92.9	62	88.6
Hispanic or Latino	–	–	8	11.4
Asian	1	3.6	3	4.3
Native Hawaiian/other	1	3.6	1	1.4
Black/African American	–	–	–	–
Other	–	–	2	2.9
Education level				
Bachelor’s	–	–	5	7.1
Master’s	26	92.9	62	88.6
Doctoral	2	7.1	3	4.3
Academic degree/background				
Marriage and family therapy	5	17.9	8	11.4
Psychology	3	10.7	4	5.7
Social work	11	39.3	19	27.1
Counseling psychology	9	32.1	28	40.0
Other	–	–	11	15.7
Primary theoretical orientation				
CBT	21	75.0	45	64.3
Family systems	6	21.4	7	10.0
Solution-focused	1	3.6	3	4.3
Humanistic	–	–	4	5.7
Psychodynamic	–	–	7	10.0
Play therapy	–	–	3	4.3
Art therapy	–	–	1	1.4
Licensed	27	96.4	36	51.4
Mainly uses EBT	21	75.0	51	72.9
	*M*	SD	*M*	SD
Age	44.4	10.4	38.0	11.5
Years providing therapy	14.1	7.6	7.0	6.2
Years at organization	10.4	6.4	4.7	4.1
Caseload size	12.6	12.1	30.1	12.6
Number of clinician supervisees	7.5	4.7	–	–
Percentage of time on supervision	36.6	18.3	–	–
Percentage of time on clinical work	26.9	20.5	–	–
Number of different types of TF-CBT training	5.0	1.8	3.9	2.0

#### Clinician participants

Clinicians were eligible for study inclusion if they were trained in TF-CBT through the EBT initiative, provided TF-CBT to children and adolescents, were supervised by one of the participating supervisors, were employed at least 80% full-time equivalent, and provided treatment in English (to enable coding of TF-CBT fidelity for other analyses). Ninety-five clinicians were enrolled in phase I; we analyzed data from the 70 (74%) who were recorded in supervision sessions.

### Measures

#### Participant characteristics

Participants provided information on their age, sex, ethnicity, race, education, licensure status, theoretical orientation, and other relevant background information (see Table 1). Supervisory-specific information was also obtained (e.g., number of supervisees, time spent supervising vs. direct clinical work). TF-CBT training was measured using a summative index from 12 training activities (e.g., in-person TF-CBT training, read published TF-CBT manual, etc.).

#### Occurrence of weekly supervision

Supervisors completed a weekly survey for the duration of the study (up to 43 weeks) reporting on whether or not supervision occurred with each study clinician and if a TF-CBT case was discussed. If supervision did not occur, we collected information on why (e.g., vacation, crisis, training, medical leave, other [write-in option]). This survey provided an indication for how many recordings we should expect to receive.

#### Supervision Process Observational Coding System

The Supervision Process Observational Coding System (SPOCS) is an adaptation of the Therapeutic Process Observational Coding System for Child Psychotherapy—Strategies scale (TPOCS-S) [38, 39]. The TPOCS-S is a coding measure for characterizing psychotherapy strategies in usual clinical care for youth, typically using video or audiotaped recordings. It includes 31 items on five theoretical orientation subscales: behavioral, cognitive, psychodynamic, client-centered, and family therapy. The TPOCS manual includes detailed descriptions and examples of each strategy, with guidance for strategy discrimination. At 5-min intervals, strategies are rated for occurrence and intensity (low, medium, or high). Ratings across intervals are used to estimate an overall intensity score per strategy (7-point Likert scale) for the entire session, which captures both frequency (number of 5-min intervals in which it occurred) and intensity (ratings within intervals).

For the current study, the TPOCS-S was used as a basis to design a coding system to capture clinical supervision of TF-CBT. The SPOCS also applies an adaptation employed by Garland et al. [40] in their use of the TPOCS-S, in which strategies were divided into therapeutic content and techniques. Our resulting coding measure for supervision, the SPOCS, included 29 supervision strategies, with 16 content areas and 13 techniques (see Additional file 1 for detailed descriptions). As audio (and not video) recordings were used for coding, coders could not code non-verbal behavior. Content included six practice elements common in many CBT-based interventions and particularly common among EBT approaches for anxiety and behavior problems: assessment, psychoeducation, coping skills, exposure, cognitive processing, and behavior management. Four items were specific to TF-CBT and/or trauma-specific treatments: client’s trauma history, preparation for conjoint parent/child sessions, creative application of TF-CBT elements, given child-focused treatment (i.e., use of art, play, and books), and trauma-related safety. Three other general clinician-level EBT techniques found to be infrequently used by clinicians in usual care [40] were included: assigning/reviewing client homework, client behavioral rehearsal, and clinician modeling in session. Two items, treatment engagement and parent-level challenges, were added due to the frequency with which they were mentioned as challenges encountered in delivering TF-CBT in community settings [41]. A final content code captured case management and other topics.

The 13 items included in the supervision techniques domain were identified through literature review [26, 42, 43], review of other supervision and consultation coding manuals [11, 44, 45], and expert consensus. Five were specifically considered gold standard techniques: symptom monitoring, reviewing actual practice (audio/video, client work produced in session), fidelity or adherence assessment, clinician behavioral rehearsal in supervision, and supervisor modeling. Supervision techniques included in the SPOCS are likely applicable to supervision of general treatment and other EBT (see full list in Fig. 2; detailed descriptions in Additional file 1).

As with the TPOCS-S, trained coders rated strategy occurrence in 5-min intervals (low, medium, or high), ultimately determining intensity scores for content and techniques for the session (0–6 range; 0: non-occurrence; 1–2: low; 3–4: medium; 5–6: high intensity). For example, a low-intensity rating on the exposure item would reflect only a brief mention (e.g., “You should start the trauma narrative”). A high-intensity rating would reflect a more detailed discussion from a past or upcoming session. A low-intensity rating of supportive listening would be given for a limited number of supervisor non-specific acknowledgements or general praise (e.g., “nice work”; “that sounds hard”), while a higher score would be given if the supervisor provided more frequent and explicit support, validation, or praise (e.g., “…sounds like a tough session; still, you did a really nice job getting this super anxious kid to feel comfortable talking about his sexual abuse. I am impressed.”). Additionally, coders tallied the number of clinical cases reviewed and the temporal focus of each 5-min interval (i.e., review of past session, planning for a future session, or both).

### Coder training/supervision session sampling, and reliability

#### Coder training

Study coders were six post-baccalaureate research assistants. All coders were first trained in coding TF-CBT fidelity reliably, a pre-requisite for being trained to code supervision of TF-CBT. Coders also attended a 2-day clinical training on TF-CBT, completed a 10-h web course, read the TF-CBT treatment manual [37], and received additional didactic training from the first and last authors in distinguishing components of the treatment model. Supervision-focused coder training included independent study of the SPOCS, didactic training, independent coding of 25 supervision sessions, and group review (led by the first author), with joint listening when necessary to reach consensus. All coders then independently coded ten training files to ensure acceptable interrater reliability across group members and with the first author. Coders began official study coding once their individual ratings reached an established criterion: interrater reliability at the overall level, intraclass correlation coefficient (ICC) _(2,1)_ ≥ .80 [46]. For any individual content/technique item with an ICC_(2,1)_ ≤ .60, coders were assigned additional review and practice. To prevent drift, coders were required to reread the coding manual monthly and attend periodic booster trainings. Supervision files were randomly assigned to each coder.

#### Session sampling procedures

We received 667 recordings across the 28 supervisors who submitted individual TF-CBT supervision sessions. We excluded 29 files shorter than 1 min (4.3%) but kept all others as they represented the supervision received, even if brief. Of the remaining 638, we coded 438 (70%). We chose to code 23 recordings per supervisor, as 23 represented a natural breakpoint in the frequency distribution of recordings received per supervisor. Ten (of 28) supervisors submitted over 23 recordings. Stratified random sampling was used to ensure distribution of recordings across time and clinicians. Eighteen (of 28) submitted fewer than 23 recordings and all were coded (*M* = 10.8; *SD* = 4.9; range 4–19).

#### Interrater reliability

Of the 438 sampled session recordings, 105 (23.9%) were coded by multiple coders to test interrater reliability. The overall group average ICC assessing reliability was ICC_(2,6)_ = .87, which represents excellent reliability [46]. Each coder had excellent individual ICCs of .84 or higher. At the item level, ICCs ranged from .28 to .96. Of note, only four individual item-level codes (out of 29) were below .60. The two in the “poor” range (< .40), cognitive processing and clinician behavioral rehearsal in session, had relatively low incidence and low variance, which can result in unreliable estimates of interrater reliability [47–49].

### Analyses

Frequencies were used to calculate the percent of sessions in each broad intensiveness category (i.e., low, medium, high). To examine the variance in content and technique intensity attributable to clinician and supervisor levels, ICCs were calculated using unconditional three-level random effects mixed models (session nested within clinician nested within supervisor, with random intercepts for clinician and supervisor).

## Results

Using the Weekly Occurrence of Supervision Survey, supervisors reported 697 supervision sessions of TF-CBT cases involving 70 clinicians. Survey responses indicated that supervision did not consistently occur each week. Supervisors submitted 638 recordings of TF-CBT supervision sessions with these 70 clinicians, resulting in an overall submission rate of 91.5%. Most clinicians (85.7%) were missing only two or fewer recordings. There were no significant differences between supervisors who submitted or did not submit recordings based on sex, race/ethnicity, highest academic degree, years providing psychotherapy, years employed at the participating organization, or self-reported use of EBT. However, those who submitted recordings were significantly older (mean age = 44.4 vs. 37.8, *p* < .05), more likely to endorse their primary theoretical orientation as CBT (75 vs. 0%, *p* < .05), and less likely to endorse family systems therapy (21 vs. 60%, *p* < .05) or art/play therapy (0 vs. 40%, *p* < .05).

Clinicians who were recorded did not significantly differ from clinicians who were not, based on sex, age, race/ethnicity, years employed at the organization, licensure status, primary theoretical orientation, or self-reported use of EBT. However, clinicians who were recorded had provided psychotherapy for more years (*M* = 7.0 vs. 4.3, *p* < .05) and were *less* likely to have a degree in Marriage and Family Therapy (11 vs. 40%, *p* < .05).

The coded sample of TF-CBT supervision sessions addressed an average of 2.1 cases per recording (*SD* = 1.6, range 1–11) and lasted an average of 26 min (*SD* = 15.0, range 1–72), resulting in an average of 12.4 min per case (*SD* = 8.6). Across supervision sessions, of the 5-min intervals coded for time orientation, 58.4% focused on both past and future session content, 32.2% focused only on the past session, and 9.5% focused only on future sessions.

### Supervision strategies

#### Supervision content

**Fig. 1:**
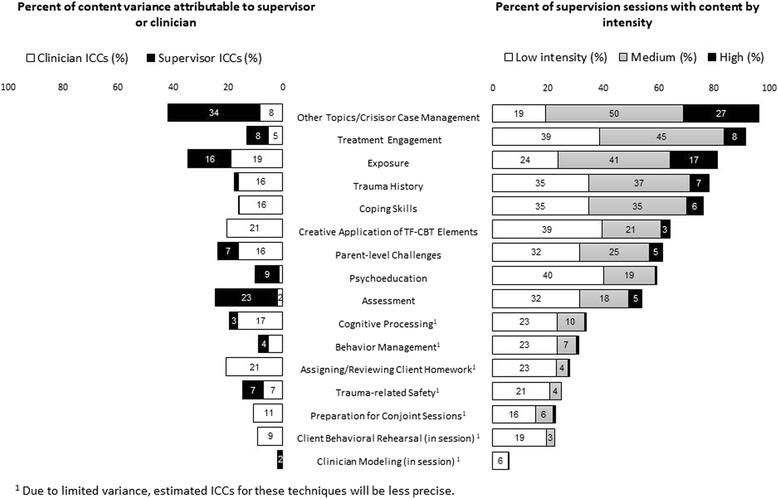
Content of EBT Supervision

The right side of Fig. 1 examines occurrence for each of the 16 content areas. For example, “other topics/crisis or case management” was not discussed at all in 3.7% of the sessions and was covered at low intensity in 19.2%, medium intensity in 49.8%, and high intensity in 27.4% of the sessions. Overall, this content area was discussed frequently (96% overall occurrence), but usually at medium intensity.

Additional content areas that occurred in more than 50% of the supervision sessions were treatment engagement (92%), exposure (81%), trauma history (78%), coping skills (76%), creative application of TF-CBT elements (64%), parent-level challenges that impact TF-CBT (62%), psychoeducation (60%), and assessment (54%). Content areas occurring in 25% or fewer of the supervision sessions were trauma-related safety (25%), preparation for conjoint sessions (23%), client behavioral rehearsal (22%), and clinician modeling (6%).

In looking at intensity of coverage, supervision content areas that occurred predominantly at low intensity included three CBT elements (psychoeducation, cognitive processing, and behavior management), two trauma treatment-specific elements (trauma-related safety, preparation for conjoint sessions), and all three EBT techniques (assigning/reviewing client homework, client behavioral rehearsal, and clinician modeling). Content areas most frequently occurring with the highest intensity included other topics/crisis or case management, exposure, treatment engagement, trauma history, and coping skills.

The left side of Fig. 1 provides ICCs for variance at the clinician and supervisor levels (i.e., the degree to which the pattern of content across supervision sessions is similar within individual clinicians and supervisors, respectively). The ICCs for items with low occurrence (e.g., clinician modeling, client behavioral rehearsal) are likely to have poorer reliability, as with any statistical estimate drawn from rare events. Items with variance attributable to the clinician level in high proportions included assigning/reviewing client homework (21%), creative application of TF-CBT elements (20%), cognitive processing (17%), parent-level challenges (16%), trauma history (16%), and coping skills (16%). Items with variance attributable to the supervisor level in high proportions included other topics/crisis or case management (34%) and assessment (23%). Variance in exposure coverage was attributable to both clinician (19%) and supervisor levels (16%).

#### Supervision techniques

**Fig. 2:**
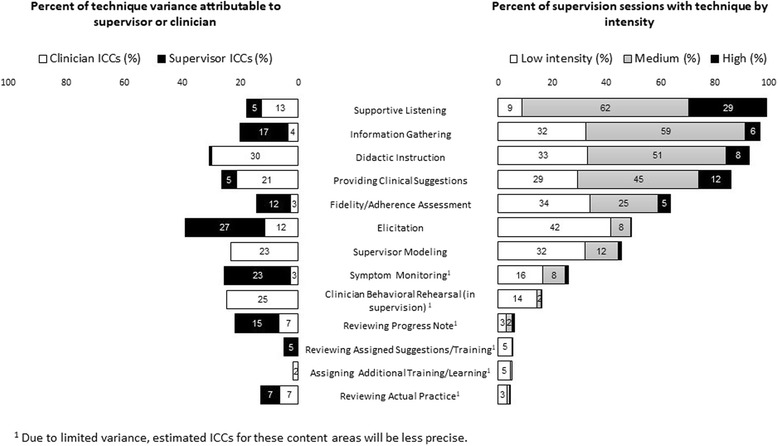
Techniques of EBT Supervision

The right side of Fig. 2 examines occurrence for each of the 13 techniques. The most frequently occurring was supportive listening, which occurred in 434 (99%) of the coded sessions. Other techniques that occurred in more than 50% of the sessions were information gathering (97%), didactic instruction (93%), providing clinical suggestions (86%), and fidelity/adherence assessment (64%). Techniques occurring in 25% or fewer sessions were clinician behavioral rehearsal in supervision (16%), progress note review (6%), reviewing actual practice (e.g., audio/videotape; reviewing in-session materials) (5%), assigning additional training/learning (5%), and reviewing assigned suggestions/training (5%).

Looking at intensity of coverage for techniques, eight were covered mostly at low intensity. These items included four of the five gold standard elements (all but fidelity/adherence check). Techniques that occurred mostly at medium or high intensity included supportive listening, information gathering, didactic instruction, and providing clinical suggestions.

The left side of Fig. 2 provides ICCs for variance at the clinician and supervisor level for supervision techniques. Techniques with variance attributable to the clinician level in high proportions included didactic instruction (30%), clinician behavioral rehearsal (25%), supervisor modeling (23%), providing clinical suggestions (21%), supportive listening (13%), and elicitation (12%). Techniques with variance attributable to the supervisor in high proportions included elicitation (27%), symptom monitoring (23%), information gathering (16%), progress note review (15%), and fidelity/adherence assessment (12%).

## Discussion

To our knowledge, this is the first study to objectively describe content and techniques used by EBT-trained, workplace-based clinical supervisors in the context of an EBT initiative. Our findings suggest that workplace-based supervision may offer a strategy to support EBT implementation, but also point to areas for enhancement, particularly in the use of gold standard techniques. These results have relevance for the broader field of implementation science, in that coding an implementation strategy (i.e., clinical supervision) in a specific practice setting (i.e., public mental health) at multiple levels (i.e., supervisor and provider) revealed some important, though perhaps unsurprising, differences from supervision in efficacy and effectiveness trials. As noted by others, defining “what works” needs to include what is “practical, feasible, and affordable, and therefore, what is effective” [40]. Below, we first note differences and then discuss supervision content and techniques.

First, the average allotted time per case was about 12 min. Although to our knowledge, time per case in efficacy and effectiveness trials is not documented in the literature, from our collective experience as investigators and supervisors in trials, we would guess that it typically exceeds the average allotted time found in the current study. Second, although not the goal of this study, our results add to other findings that individual supervision may not occur on a weekly basis in public mental health [18, 50], despite being described by organizations as a common infrastructure support [16]. If supervision does not occur weekly, it may mean that cases have to be discussed with more limited time. Third, compared to efficacy trials [27], workplace-based clinical supervision of EBT was rarely informed by reviewing actual practice.

In the average 12 min per case, supervision regularly covered treatment engagement and parent-level challenges (in 50% or more sessions; often at medium or high intensity), potentially leaving limited time to intensely cover EBT content. The most commonly and intensely discussed EBT content was exposure, which converges with objective coding of anxiety-focused expert consultation [11] and our experiences in TF-CBT efficacy and effectiveness trials. This is promising, as clinicians in public mental health may rarely use exposure [39], potentially due to limited training and feeling less comfortable with this practice element [51]. It remains an empirical question—and one we hope to answer—whether coverage in supervision is related to exposure use in client sessions. Variance in coverage of exposure was attributable to *both* clinician and supervisor levels, suggesting that certain clinicians and certain supervisors more consistently review exposure.

Conversely, other EBT content, including behavior management skills, cognitive processing, and clinician EBT techniques (i.e., assigning/reviewing client homework, clinician modeling in session) were infrequently discussed. Behavior management skills and cognitive processing are reported as challenging for clinicians [41, 52] and likely need more attention in supervision, particularly given the high comorbidity of behavioral problems in public mental health [53]. Garland and colleagues’ work [40] in usual care indicates that clinicians rarely use EBT techniques, and our work shows that they also are rarely discussed in supervision. Interestingly, our findings of infrequent coverage diverge from Accurso and colleagues’ study [26] in which EBT techniques were reportedly discussed frequently in supervision. Divergence may be due to their study focus (trainees vs. staff) or to different methods (self-report vs. coded interactions).

Turning to techniques, two gold standard techniques occurred frequently and often at medium (but not high) intensity. Fidelity assessment occurred in more than half of the sessions. Symptom monitoring was used in nearly half, possibly due to the longstanding focus on assessment in the Washington EBT initiative [54]. Our coders anecdotally reported that supervisors did not seem to use formal checklists to monitor fidelity, but did informally inquire about upcoming TF-CBT elements and discussed the treatment model as it applied to a case, beyond merely planning for the next session. This level of fidelity monitoring may be appropriate, given the field’s interest in considering both effective and efficient methods [55, 56] and specific constraints (e.g., higher caseloads, less time in supervision) in public mental health.

Three gold standard supervision techniques were infrequently used and/or used mostly at low intensity. These included reviewing actual practice, clinician behavioral rehearsal in supervision, and supervisor modeling. These findings also diverge from previous self-report studies. For example, our rates of reviewing actual practice are substantially lower than those from both a national survey of community mental health (albeit nearly 10 years ago) in which nearly 20% reported audio or videotape review [16] and are lower than the 13% reported in Accurso’s study [26]. Our findings provide some confirmation that audio and videotape review, a commonly employed technique in treatment trials, may not be feasible for many community settings [57]. Given that some organizations may be too resource-constrained to even provide individual supervision, or may provide individual supervision only to trainees or unlicensed clinicians, implementation efforts that expect regular audio/videotape review likely represent a substantial change in usual practice. Interestingly, our rates of reviewing actual practice are low (5%) and still may overestimate audio/videotape use, as our code included review of in-session materials (e.g., child’s trauma narrative) due to their practicality and potential promise as methods of fidelity assessment [58].

Behavioral rehearsal—identified in expert consultation as a technique that may lead to better fidelity for some clinicians [11, 14] and as a potentially efficient method of assessing *analogue* fidelity [59]—was rarely used and almost always at low intensity. Supervisor modeling, in contrast, was used in nearly half of the supervision sessions, but at low intensity. Why behavioral rehearsal and modeling were rarely used, or used at very low intensity, is unclear. Possibly, behavioral rehearsal may cause nervousness [11], leading to lower use. Alternatively, these techniques, although common in efficacy trials [27], may be less common in training programs and disciplines of many public mental health supervisors and clinicians (e.g., Social Work), where a greater focus may be on clinical process vs. clinical content. Limited supervision time may also play a role, as discussion may be deemed more expedient.

The degree to which the use of content and techniques of supervision clustered at the clinician or supervisor level varied. Content items were more likely to cluster at the clinician level, while technique items were equally likely to cluster at either level. It may be that clinician-level characteristics (e.g., EBT experience, skill) are more likely to drive *content* of supervision, with *techniques* driven somewhat equally by clinician and supervisor characteristics. Across all content and technique items, other topics/crisis or case management clustered the most at the supervisor level. Interestingly, three of the four techniques that clustered at the clinician level are focused on methods of teaching (i.e., didactic instruction, clinical suggestions, and modeling), suggesting that certain clinicians may need more instruction during supervision.

Techniques conceptualized as gold standard did not consistently cluster at either level (i.e., proportionally, supervisor modeling clustered mostly within clinician, while symptom monitoring and fidelity assessment clustered mostly within supervisor) or occurred so infrequently that clustering estimates may be unreliable (i.e., clinician behavioral rehearsal, reviewing actual practice). As might be expected, our results suggest that supervision is tailored to individual clinicians, or equally as likely—individual clinicians “pull” for different things in supervision. They also suggest that supervisors may have a style, in which some techniques are used more consistently than others. Our team is currently investigating individual clinician, supervisor, and organizational characteristics that may predict content coverage and technique use, the interplay between the two, and whether as in the audit and feedback literature from the medical field [29], certain types of clinicians benefit more from specific techniques (i.e., moderators of implementation strategy effectiveness).

We also examined the temporal focus of supervision, as in our experience, supervision in efficacy and effectiveness trials typically includes a strong focus on planning for upcoming sessions. Due to our coding method (i.e., 5-min intervals), sensitivity was limited. Still, findings suggest that supervision was focused more on past sessions, which is necessary for evaluating fidelity, determining any needed “course corrections,” and the starting point for the next session. However, an over-focus on the past may fall short in providing the necessary support for clinicians to effectively deliver EBT. Techniques like supervisor modeling and clinician behavioral rehearsal are most likely deployed when discussing future sessions, and in our sample, these techniques and future sessions received less focus.

Some limitations should be considered. First, we did not collect data on supervisors who chose not to participate. Second, four supervisors reported supervising few TF-CBT cases and submitted only four or five recordings; these recordings may not be representative. Third, our coding protocol captured extensiveness of coverage, but not consistency with the EBT (e.g., were clinical recommendations appropriate, per TF-CBT). Anecdotally, coders’ reported that recommendations were aligned with TF-CBT fidelity, but this was not empirically evaluated. Knowing whether supervisors contribute to or protect against EBT drift would be a beneficial aspect of future coding efforts. Fourth, given that we coded audio recordings, nonverbal interactions could not be captured. Fifth, we could not examine clustering at the organizational-level due to few having more than one participating supervisor. Finally, without guidelines from the empirical literature (i.e., efficacy or effectiveness trials), we cannot comment on what levels of extensiveness are necessary for clinician fidelity.

It is important to note that supervisors in our sample participated in a state-funded EBT initiative and had access to a range of supervisor-specific supports (described earlier), including some training in gold standard techniques [23]. However, our findings support those from a small RCT that found that workshops alone may not be enough to change practice among supervisors [60], paralleling findings from clinician training studies. Our team is currently completing a RCT in which supervisors received training plus ongoing support and monitoring in integrating specific gold standard techniques into supervision [17]. The goal is to determine if routine use of gold standard techniques might impact clinician EBT fidelity and downstream client outcomes.

## Conclusions

Increasingly, implementation efforts need to advance beyond examining practices with providers and clients to examining “real-world supervisors and managers” [7]. With some exceptions [61], supervisors seem to have longer tenure at their organizations [18] and many organizations support some form of workplace-based supervision. To leverage workplace-based supervision, however, the field requires “a better understanding of how supervisors should be trained and included in the implementation process” [3]. We see our study as an important step towards describing workplace-based clinical supervision of EBT in public mental health. We also see our study as an example of how objective coding of implementation strategy use in usual care settings (vs. relying on self-report) can inform our understanding of specific discrepancies from efficacy trials that might impact provider practice. Objective coding methods may allow for better accuracy in identifying moderators and mediators of implementation outcomes, even further advancing the potential impact of implementation science.

## Additional file

Additional file 1: Supervision Process Observational Coding System (SPOCS): Content and Technique Domains. (DOCX 35 kb)

## Abbreviations

EBT: Evidence-based treatment
ICC: Intraclass correlation
MST: Multisystemic therapy
SPOCS: Supervision Process Observational Coding System
TF-CBT: Trauma-focused Cognitive Behavioral Therapy
TPOCS-S: Therapeutic Process Observational Coding System for Child Psychotherapy—Strategies Scale
