# Supplementary material for: Objective coding of content and techniques in workplace-based supervision of an EBT in public mental health
Source: Implement Sci. 2018 Jan 24;13:19. doi: 10.1186/s13012-017-0708-3 (PMC5784597; doi:10.1186/s13012-017-0708-3)
Supplement: Additional file 1: — Supervision Process Observational Coding System (SPOCS): Content and Technique Domains. (DOCX 35 kb) [file 13012_2017_708_MOESM1_ESM.docx]

Additional File 1. Supervision Process Observational Coding System (SPOCS): Content and Technique Domains

| Brief Definition & Example(s) | |  |
| --- | --- | --- |
| **Content** | |  |
| Trauma History ^Δ^ | Discuss information about the child's trauma history, e.g., abuse reporting, child welfare experiences related to the trauma, or ongoing contact with a perpetrator(s).  *Examples*:   - Clinician: “He was sexually abused for about 6 years; I think he saw a lot of family violence.”^1^ - Supervisor: “Did she see her dad get killed? Sounds like that isn’t clear.” | |
| Treatment Engagement | Discuss the child and/or caregiver(s’) treatment engagement, involvement, attendance, and/or participation  *Example*:   - Supervisor: “Is mom coming weekly now or not? I know you were having trouble with no-shows.”^2^ | |
| Assessment ^ψ^ | Discuss the child’s psychiatric symptoms and/or behavior problems using standardized measures and/or functional analysis.  *Example*:   - Supervisor: “I see you have administered two assessments. On the SCARED, her score is an 8 out of 8 for PTSD…” | |
| Other Topics/Crisis or Case Management | Discuss topics that do not reflect the other content items, e.g., issues unrelated to the child’s traumatic experiences and/or not directly related to TF-CBT components.  *Examples*:   - Clinician: “The CPS worker is saying she doesn’t want to take her medication.” - Supervisor: “I have your benefit accrual, if you want to see that.” | |
| Psychoeducation ^ψ^ | Discuss Psychoeducation, e.g., teaching the client about typical reactions to trauma, trauma prevalence, and/or providing an overview of TF-CBT treatment.  *Example*:   - Clinician: “We talked about some of the common responses to trauma, and normalized her own symptoms.” | |
| Behavior Management ^ψ^ | Discuss Parenting Skills, e.g., praise, active ignoring, rewards, and/or consequences.  *Example*:   - Clinician: “Her mom’s using the sticker chart we made at home so she can get points for rewards.” | |
| Parent-level Challenges | Discuss caregiver-related issues that can impinge on and/or complicate the clinician’s ability to deliver TF-CBT and/or the parent’s ability to engage in TF-CBT.  *Example*:   - Clinician: “It’s still really hard for her mom – her mom has a trauma history herself, so hearing about the sexual abuse is triggering for her.” | |
| Coping Skills ^ψ^ | Discuss the TF-CBT coping skills, which could include: relaxation, affective identification and/or modulation, and cognitive coping.  *Example*:   - Clinician: “We practiced some belly breathing. Then we talked about what sadness looks like and how it feels in your body.” | |
| Exposure ^ψ^ | Discuss and/or plan for the trauma narrative, *In Vivo* exposure, and/or gradual exposure to the child’s trauma with the child and/or caregiver(s).  *Example*:   - Supervisor: “Do you have a sense of whether or not you want to have her write out her narrative first and then add thoughts and feelings? Or do you want to do that as you go?” | |
| Cognitive Processing ^ψ^ | Discuss using cognitive processing techniques for changing unhelpful and/or untrue thoughts about the trauma, e.g., responsibility pie or best friend role-play.  *Example*:   - Clinician: “She has told herself, ‘I can never show weakness, I can never be vulnerable,’ and I think helping her explore more helpful thoughts around the trauma might help…” | |
| Preparation for Conjoint Sessions ^Δ^ | Discuss preparing the child and/or caregiver(s) and/or other adults in the child’s life who will be participating in conjoint TF-CBT work for future conjoint sessions, e.g., sharing the trauma narrative.  *Example*:   - Supervisor: “I would go ahead and meet with the parents and go through the whole narrative with them ahead of time so they can actually get some of their own feelings out with you.” | |
| Trauma-related Safety ^Δ^ | Discuss preparing children to be safe in the face of possible future dangers, including those similar to the child’s personal trauma experience and other possible dangers in the child’s life, e.g., community violence, body safety, etc.  *Example*:   - Clinician: “I talked about how we’re going to be doing safety planning, and how to have a good safe relationship, such as whether it is safe to hold hands or kiss.” | |
| Client Behavioral Rehearsal (in session) | Discuss having the child and/or caregiver(s) practice a skill in the therapy session.  *Example*:   - Supervisor: “His anxiety at home might be a really good role play to do with him to practice his emotion regulation.” | |
| Assigning/Reviewing Client Homework | Discuss homework assignment(s) for an upcoming client session or how to review homework assigned to the child and/or caregiver(s) in a prior session.  *Example*:   - Supervisor: “If she comes in and hasn’t done her homework, what would you do?” | |
| Clinician Modeling (in session) | Discuss the clinician modeling (i.e., enacting or demonstrating) a skill for the child and/or caregiver(s) in the therapy session.  *Example*:   - Clinician: “As you suggested, I helped to show him how to say ‘no’ while standing up straight, making eye contact, and using a firm voice.” | |
| Creative Application of TF-CBT Elements ^Δ^ | Discuss the use of books, computerized applications, music, games, art, and/or other play to teach and/or implement a TF-CBT component (not as a reward or as an activity separate from treatment).  *Example*:   - Clinician: “I was thinking about reading the book A Terrible Thing Happened to her.” | |
| **Techniques** |  | |
| Symptom Monitoring | Supervisor and/or clinician discuss repeated use of standardized assessment measures to determine how the child and/or caregiver(s)’ symptoms look currently and how they have changed over time, e.g., to examine treatment effectiveness.  *Example*:   - Supervisor: “So tell me again, he’s at a 13 now, what was his score on the CPSS when you started?” | |
| Progress Note Review | Supervisor reviews the progress note with clinician or alludes to review that occurred before the supervision meeting.  *Example*:   - Supervisor: "Let's look at your case note for that session”. | |
| Providing Clinical Suggestions | Supervisor gives specific ideas, suggestions, and/or directions to clinician about what to do in a future session and/or about what clinician should have done in a past session.  *Example*:   - Supervisor: “You said that last session the mom asked about what happens at court. I might have walked through the process or given her ideas for how to find out more information.” | |
| Assigning Additional Training/Learning | Supervisor makes a clear request for clinician to obtain additional training or expertise for his/her own learning (does not have to directly benefit the case being discussed).  *Example*:   - Supervisor: "There's a chapter in the new TF-CBT book on doing TF-CBT with kids in foster care. You should check that out and see if there are any ideas for this client.” | |
| Reviewing Assigned Suggestions/Training | Supervisor specifically checks in about and/or asks about a suggestion, strategy, training, and/or other recommendation from a past supervision session.  *Example*:   - Supervisor: “So, help me understand, it sounds like you didn’t sign up for the CBT+ training like we talked about. What happened with that?” | |
| Review of Actual Practice | Prior to or during supervision, the supervisor either: a) watched or listened to a recording of clinician’s actual clinical practice with a client or b) reviewed client work from a past TF-CBT session, e.g., reviews the child’s trauma narrative, looks at triangles the child and/or caregiver(s) created during cognitive coping.  *Example*:   - Supervisor: “Watching part of your tape, I noticed that you did most of the talking throughout the session. Tell me why that might not be the most effective way to change the mother’s behavior with her child.” | |
| Fidelity/Adherence Assessment | Supervisor and/or clinician discuss the topic of fidelity to TF-CBT or clinician’s progress through the model.  *Example*:   - Supervisor: “As you know, PRACTICE is an acronym for the TF-CBT components. Tell me which components you have completed and which one you’re on now.” | |
| Elicitation | Supervisor uses questions to a) encourage/elicit clinician thinking and planning for a subsequent session (as opposed to providing ideas/suggestions) or b) help clinician evaluate his/her own effectiveness in a past session.  *Example*:   - Supervisor: “He’s really blaming himself for what happened. What do you think are some possible other ways to view the situation, given what you know about the case?” | |
| Didactic Instruction | Supervisor provides information, teaches, and/or explains something to clinician via "lecture" or in a didactic style.  *Example*:   - Supervisor: “There is research showing that sometimes PTSD symptoms can sound like hallucinations, for example thinking they can hear the offender talking.” | |
| Supportive Listening | Supervisor makes statements that reflect, validate, acknowledge, and/or praise clinician.  *Example*:   - Supervisor: “Sounds like a frustrating situation." | |
| Clinician Behavioral Rehearsal (in supervision) | Supervisor guides clinician through practicing effective use of a therapeutic skill or technique for a future session; clinician actively practices a skill playing the role of the therapist.  *Example*:   - Supervisor: “Okay, let me just pretend to be the kid—‘but it’s too hard! I can’t talk about what happened.’” [Clinician proceeds to play the clinician role and respond] | |
| Supervisor Modeling | Supervisor models (i.e., enacts or demonstrates) a specific clinical skill or method of delivering a treatment component.  *Example*:   - Supervisor: “You might say, ‘Hey, is there anything that happened in your past that guides how you deal with your daughter?’” | |
| Information Gathering | Supervisor gathers information about the case, a past session, and/or clinician’s therapeutic/TF-CBT skill-level, e.g., asking the clinician for information about the child, child’s symptoms, family’s problems, and/or context, e.g., living environment.  *Example*:   - Supervisor: “When is the IEP meeting?” | |

^1^Supervisor or clinician language was coded in supervision.

^2^Many examples, including this one, were co-coded with multiple content and/or technique items; please see the manual for guidelines and coding, available upon request from the first author.

ψ Content items common in many CBT-based EBTs, especially those for anxiety and behavior problems.

Δ Content items that are trauma-specific.
